# Quantitative Analysis of Ionic Channel Network Variation in Nafion Under Continuous Annealing Using Current-Sensing Atomic Force Microscopy

**DOI:** 10.3390/polym18101204

**Published:** 2026-05-15

**Authors:** Osung Kwon, Byungrak Son

**Affiliations:** 1 Faculty of Science, Tabula Rasa College, Keimyung University, Daegu 42601, Republic of Korea; 11502@gw.kmu.ac.kr; 2 Division of Energy & Environmental Technology, DGIST, Daegu 42988, Republic of Korea

**Keywords:** proton exchange membrane fuel cell, proton exchange membrane, ionic channel network, current-sensing atomic force microscopy, numerical approximation method

## Abstract

Proton exchange membranes (PEMs) are essential for PEM fuel cells, with proton conductivity arising from the hydration-induced ionic channel network. PEM performance can be enhanced through pretreatments, such as annealing, which reconstruct the ionic channels. This study investigates the ionic channel network variation in Nafion 212 under continuous annealing at 90 °C using current-sensing atomic force microscopy (CSAFM). A nanoscale PEM fuel cell was formed with a Pt-coated CSAFM tip and Pt-coated Nafion surface. Topography and surface roughness analyses revealed geometrical changes from annealing. Current-sensing images and histograms qualitatively assessed local conductance and ionic channel distribution. The ionic channel network density was quantitatively evaluated using the number of protons moving through the ionic channel network (NPMI), derived from CSAFM and electrodynamics principles. NPMI directly reflects ionic channel density. From the unannealed state to 60 h, NPMI increased linearly at 1 × 10^4^ h^−1^, indicating enhanced channel formation. Beyond 60 h, NPMI decreased linearly at 1.9 × 10^5^ h^−1^, reflecting progressive network degradation. As the ionic channel network increases, the number of protons reaching the membrane surface also increases, whereas in the opposite case it decreases. Thus, NPMI becomes evaluation criterion for ionic channel network density. These findings systematically link nanoscale structural changes to ionic channel reconstruction and proton transport in Nafion 212, providing insight into PEM performance evolution under thermal treatment.

## 1. Introduction

Proton-exchange membrane fuel cells (PEMFCs) are a promising solution to global warming, as they convert chemical energy directly into electrical energy without carbon dioxide emissions. PEMFCs exhibit high energy-conversion efficiency, high energy density, and zero carbon dioxide emissions [1]. Theoretical PEMFC efficiency is 83.1% under standard state (25 °C, 1 atm) [2]. Efficiency of commercialized PEMFC is approximately 45–60% [3,4]. Faseeh Abdulrahman et al. reported that energy densities of PEMFC are approximately 5–10 times larger than Li-ion battery [5,6]. Owing to the growing demand for green energy technologies, several companies have attempted to commercialize PEMFCs [7,8,9]. Because PEMFCs can generate electrical power ranging from a few watts to several hundred kilowatts, they are suitable for diverse applications, including power plants, microgrids, and portable power systems [7,8,9].

Among the various components of PEMFCs, proton exchange membranes (PEMs) are critical, serving as gas separators, anode–cathode insulators, and proton conductors [10]. Consequently, PEMs directly influence the performance and reliability of PEMFCs. Among the available PEMs, Nafion is one of the most widely commercialized materials due to its favorable chemical stability, mechanical strength, and proton conductivity [11]. These properties arise from its unique morphology. Nafion consists of a hydrophobic polytetrafluoroethylene (PTFE) backbone and hydrophilic sulfonic acid side chains, which form numerous hydrophilic channels for proton transport. By hydration, hydrophilic functional groups (-OH, -SO_3_H) absorb water and expand the volume of the channels. Furthermore, the distributed hydrophilic domains are connected to each other and continuous water channels are created. Thus, the proton conductivity of the membrane is increased [12]. As a result, Nafion exhibits phase-separated morphology with distinct hydrophilic and hydrophobic domains. The hydrophobic domains provide mechanical, chemical, and thermal stability, whereas the hydrophilic domains facilitate proton conductivity.

**Figure 1:**
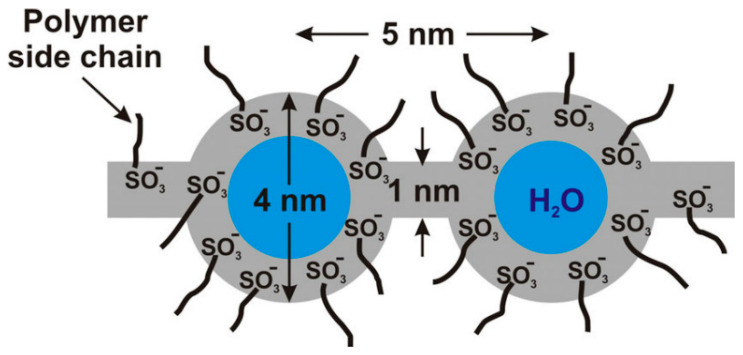
Schematic representation of the cluster network model of Nafion [13].

Understanding the morphology of Nafion is essential for determining appropriate operating conditions and improving performance; however, this remains challenging due to its complex nanoscale structure. In addition, the morphology depends strongly on temperature and water content. Numerous studies have investigated the morphological structure of Nafion. Mauritz et al. [13] were among the first to examine its morphology, proposing a cluster network model based on small-angle X-ray scattering (SAXS) and wide-angle X-ray scattering (WAXS). In this model, ionic clusters with an inverted micellar structure are spherical, with a diameter of approximately 4 nm. These clusters are interconnected by water channels approximately 1 nm in width (Figure 1), forming an ionic channel network. The development of this network depends on the water content of Nafion. Protons are transported through the ionic channel network; therefore, proton conductivity is directly related to its development.

**Figure 2:**
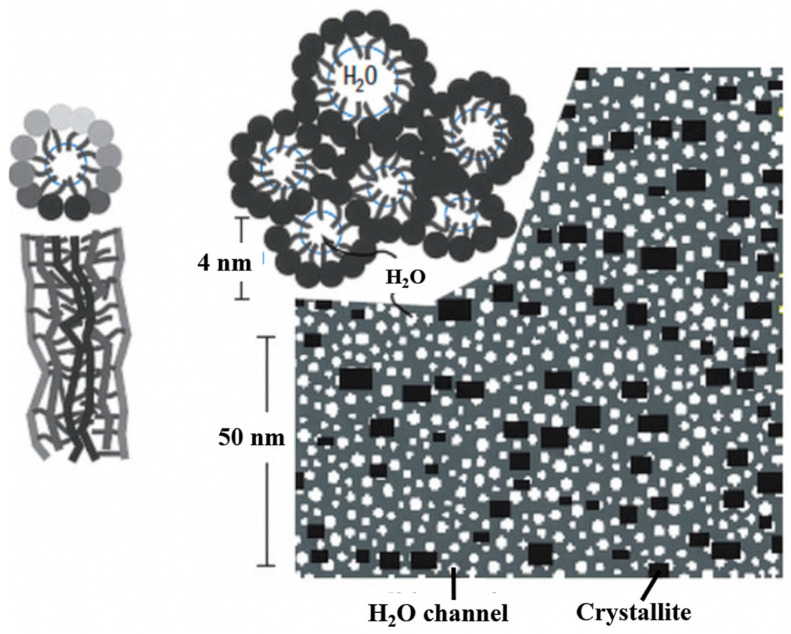
Schematic of the cylindrical water channel model of Nafion [14].

Schmidt-Rohr and Chen [14] proposed a novel morphological model of Nafion referred to as the cylindrical water channel model as shown in Figure 2. Using scattering data from previous studies, they performed simulations to characterize its morphology. This model accounts for both the high proton conductivity and favorable mechanical properties of Nafion. In this framework, hydrated Nafion contains cylindrical water channels with radii of approximately 2–3 nm, which serve as proton transport pathways. The volume of these channels increases with the water content of Nafion. The relatively large channel radius predicted by the model supports efficient proton conduction. Additionally, nanoscale crystallites are randomly distributed within the membrane, contributing to its mechanical strength.

**Figure 3:**
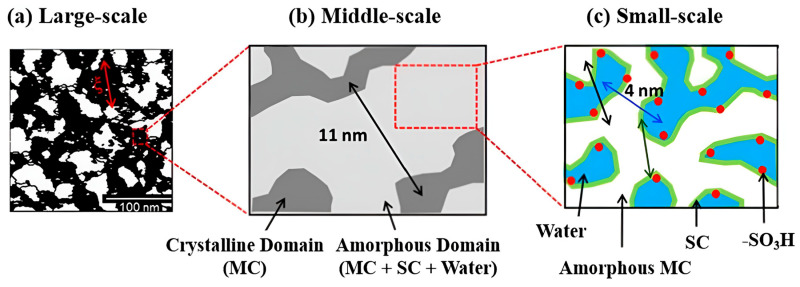
Schematic representation of the three-component domains in fully hydrated Nafion [15].

Zhao et al. [15] proposed a morphological model of the hydrated Nafion membrane based on small-angle neutron scattering (SANS). According to this study, fully hydrated Nafion consists of the PTFE-like backbone, the fluorinated side chains, and the absorbed water. The backbone and side chains exhibit a non-uniform distribution; however, water is uniformly distributed, thereby facilitating high proton conductivity. Every component of Nafion creates a bicontinuous-like structure and their mean separation distance is 11 nm as shown in Figure 3. Water and side chains are strongly interconnected, forming continuous water channels with a mean separation distance of 4 nm as illustrated in Figure 3.

Although extensive studies have investigated the morphological structure of Nafion—including the cluster network, cylindrical water channel models, and three-component domain mode–characterizing the ionic channel network remains challenging because the morphology varies dynamically with hydration, temperature, and operating conditions. Therefore, in situ characterization is required to accurately probe Nafion morphology.

Atomic force microscopy (AFM) is a powerful technique for characterizing membrane morphology at the nanoscale. In AFM, a cantilever with an ultrasharp tip scans the sample surface, and surface properties are obtained from cantilever deflection [16]. In addition to surface morphology, AFM can probe mechanical, electrical, and magnetic properties through various operational modes. Among these, current-sensing AFM (CSAFM) is widely used to investigate spatial variations in ionic properties at surfaces.

**Figure 4:**
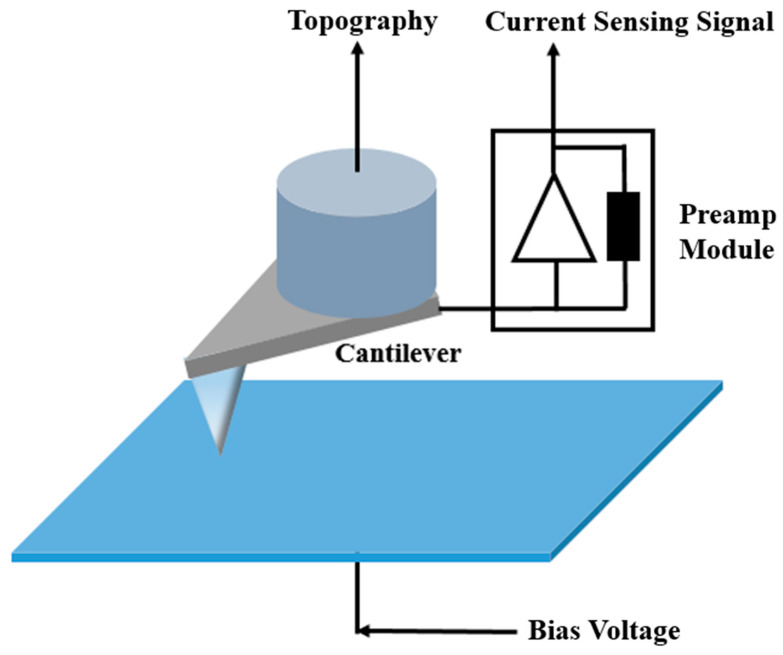
Schematic illustration of the current-sensing atomic force microscopy.

CSAFM enables simultaneous acquisition of surface morphology and local conductance images using a conductive tip [17]. CSAFM is based on conventional AFM integrated with a sensitive current measurement circuit, in which the conductive tip and sample are connected through a preamplifier within the AFM control system (Figure 4). The conductive tip is fabricated by coating a thin, uniform metal layer onto a silicon tip, allowing it to function as a current sensor. During contact-mode scanning, the tip tracks surface topography while simultaneously measuring the weak current between the tip–sample contact and a planar electrode located at the bottom of the sample. This approach enables concurrent mapping of surface morphology and local conductive distribution at the microscale.

CSAFM is a powerful technique for characterizing local conductive properties by measuring current variations across a sample surface. It offers significant potential for analyzing ionic transport in PEMs. Several studies have employed CSAFM to investigate PEM morphology [18,19]. Wang et al. [18] examined the three-phase boundary near ionic clusters using CSAFM combined with electrochemical impedance spectroscopy. They measured the local proton transport resistance under varying relative humidity (RH) conditions and observed spatial heterogeneity in proton transport resistance associated with hydrophilic regions. He and Ren [19] investigated ion conductance distribution on the surface of an anion-exchange membrane (AEM) using CSAFM. Ion conductance is defined as the mobility of ions within a specific medium. They reported a discrepancy between proton-exchange capacity and measured ionic conductance: the proton-exchange capacity was more than one order of magnitude higher than the ionic conductance under fully hydrated conditions, which was attributed to rate-limiting transport processes in the AEM. They also reported that the opposite trend occurred at 45% RH. Heisgen et al. [20] reported correlations between conductivity and mechanical properties of PEMs. Conductivity is defined as the ability of a material to conduct electricity. Surface conductance and mechanical properties were mapped using CSAFM and non-contact mode AFM, respectively. Furthermore, the hydrated surface was analyzed in terms of hydrophilic ionic clusters and current flow within these clusters. Hara et al. [21] investigated conductive regions in PEMs using CSAFM. They reported both reversible and irreversible changes in conductive areas, attributed to rearrangement of proton-conducting pathways and associated performance variations.

These studies provide important insights into the morphological structure of PEMs; however, they primarily focus on the quantitative characterization of ionic cluster networks based on morphology and on the relationship between ionic structure and surface phase distribution under varying RH conditions. Consequently, they provide limited quantitative information on the ionic structure of PEMs using CSAFM. To address this limitation, several studies have employed electrostatic force microscopy (EFM), another AFM-based technique, to investigate the ionic structure of Nafion. EFM is primarily used to characterize surface charge distribution. In this technique, a conductive, oscillating tip interacts with surface charges via electrostatic forces. EFM is therefore well-suited for characterizing electrically heterogeneous materials. In addition, numerical models have been developed to analyze EFM images and quantify the surface charge distribution in PEMs.

Barnes et al. [22] imaged ionic channel connectivity in phosphonium-containing diblock copolymer AEMs and suggested that membrane performance is directly correlated with ionic connectivity. The ionic channel morphology was quantitatively analyzed based on EFM signals. Distinct channel alignments were observed at different ion exchange capacities (IECs). At IEC = 0.44 mmol/g, cylindrical channels were aligned parallel to the membrane surface, and disconnected regions were evident. At IEC = 0.87 mmol/g, channels were oriented perpendicular to the surface, resulting in a well-connected ionic phase throughout the membrane. Yi et al. [23] synthesized a PEM based on sulfonated poly(ether ketone) and imidazolium-type ionic liquids for high-temperature PEMFC applications. The ionic structure was characterized using AFM and EFM, revealing clear phase separation between hydrophobic and hydrophilic domains under stable operation at approximately 349 °C.

Several studies have employed CSAFM and EFM to investigate the ionic structure of PEMs [18,24,25]. These studies pursue several objectives. First, a detailed understanding of the ionic channel network is required to elucidate proton conduction mechanisms in PEMs. Second, such understanding is essential for improving PEM performance. Third, insight into proton conduction mechanisms is necessary for the development of advanced PEM materials. Despite significant progress, the application of CSAFM and EFM remains insufficient for fully characterizing the ionic channel network of PEMs, as these techniques primarily probe network variations under ambient and hydrated conditions.

The operating temperature of PEMFCs is typically below 80 °C. At temperatures exceeding 80 °C, water within the PEM tends to evaporate, leading to membrane dehydration [26]. As a result, PEM performance decreases significantly. In addition, thermal degradation can be initiated by structural changes in the ionic clusters, reducing membrane lifetime. Therefore, effective thermal management is essential for maintaining PEMFC performance and reliability. During operation, PEMFCs continuously generate heat, which is regulated by the cooling system. Repeated heating and cooling cycles impose thermal stress on the PEM, leading to localized thermal degradation and eventual disruption of the ionic clusters. Minimizing thermal degradation is therefore critical for ensuring the reliability of PEM; however, this remains challenging because degradation is closely associated with thermal decomposition of ionic clusters.

Commercial Nafion typically undergoes pretreatment processes, such as annealing and other thermal treatment, to enhance its performance [27,28]. Performance improvements at approximately 100 °C are well documented and are attributed to increased ionic channel density resulting from polymer chain reorganization and increased crystallinity [26,29,30]. Under appropriate annealing conditions, both electrical and mechanical properties are improved [26,29]; however, prolonged annealing can lead to performance degradation [26,29]. Barique et al. [26] investigated proton conductivity variation in Nafion at elevated temperatures (80–100 °C) using Fourier transform infrared spectroscopy. They correlated conductivity changes with the states of ether groups and water in ionic channels, proposing that conductivity enhancement arises from an increased fraction of disordered water, which promotes the formation of hydrogen-bonded networks under annealed conditions at RH above 60%. Hensley et al. [29] examined the effects of thermal annealing on commercial Nafion. They reported increases in water permeability, proton conductivity, and equilibrium water uptake in thin membranes upon annealing. Water and proton self-diffusion were also enhanced, attributed to microstructural changes in polymer morphology. In contrast, these improvements were not observed in thicker membranes. Additionally, annealing at 165 °C resulted in a clear increase in crystallite and ionic cluster size.

Lyulin et al. [30] investigated the structure and proton conductivity of Nafion under annealing conditions using fully atomistic classical molecular dynamics simulations at various hydration levels, annealing temperatures, and cooling rates. They demonstrated that annealing induces compression of hydrophobic domains by enlarged water clusters. With increasing annealing time and cooling rate, water clusters become larger and more disconnected. Consequently, water and hydronium diffusivities decrease, leading to reduced proton conductivity. Li et al. [31] examined the durability and performance of Nafion under annealing. They reported that mechanical properties improved at temperatures below 270 °C; however, the proton conductivity of the H^+^-form Nafion decreased at annealing temperatures below 160 °C.

Understanding structural variations in the ionic channel network of PEMs is essential for improving performance and ensuring reliability. A comprehensive characterization of network evolution under continuous annealing requires high-resolution microscopic measurement, in situ observation, and quantitative analysis supported by numerical modeling. CSAFM is well suited to these requirements, as it enables simultaneous measurement of surface morphology and local current at a nanometer-scale resolution. Using a Pt-coated tip and a half membrane electrode assembly, consisting of a Pt/C-coated PEM on one side, a nanoscale PEMFC configuration can be established and probed under operating conditions. Owing to these capabilities, CSAFM allows accurate and in situ characterization of ionic channel networks in PEMs. However, numerical models for interpreting CSAFM signals in PEMs remain limited. Therefore, the development of a robust numerical model for CSAFM signal analysis would significantly enhance its utility for quantitative characterization of ionic channel networks in PEMs.

In this study, we investigate the development of the ionic channel network in Nafion 212 under continuous annealing near operating temperatures to provide guidelines for improving performance and ensuring reliability. The evolution of the ionic channel network is systematically analyzed using CSAFM. The study is conducted in several steps. First, the surface morphology and conductance maps are obtained simultaneously using CSAFM. Second, the morphological changes under continuous annealing are quantified using root mean square (RMS) roughness. Third, variations in the conductance maps are analyzed using histogram peak values and the full width at half maximum (FWHM). Finally, changes in the ionic channel network are evaluated using the numerical approximation method that estimates the number of protons moving through the network (NPMI).

## 2. Materials and Methods

**Figure 5:**
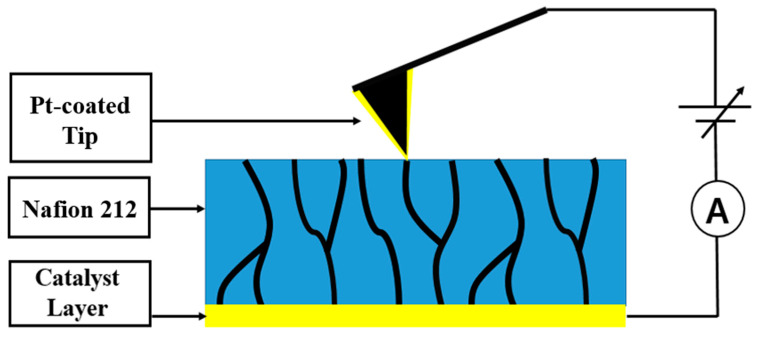
Nanoscale PEMFC configuration using CSAFM.

In this study, CSAFM was employed to measure membrane morphology and current distribution. To probe microscopic current flow in Nafion 212 (Nafion, Tyrone, PA, USA), a nanoscale PEMFC configuration was constructed using CSAFM, which is Agilent Picoscan CSAFM (Agilent, Santa Clara, CA, USA), as shown in Figure 5 [32]. A Pt-coated tip attached to the cantilever was used to form a nanoscale catalyst layer and function as the anode. A catalyst layer consisting of platinum-supported carbon (Pt/C) was applied to one side of the Nafion 212 membrane to serve as the cathode. When the Pt-coated tip contacts the Pt/C-coated Nafion 212, a nanoscale PEMFC is established. If the tip is positioned over ionic clusters, the local electrochemical reaction is activated, and the resulting current flow through ionic channels is measured as local conductance. Pt has good physicochemical characteristics such as a high melting point and excellent electrical and thermal conductivity. In addition, it has excellent chemical stability and corrosion resistance. Thus, it is used as an ideal catalyst and electrode material in fuel cells.

**Figure 6:**
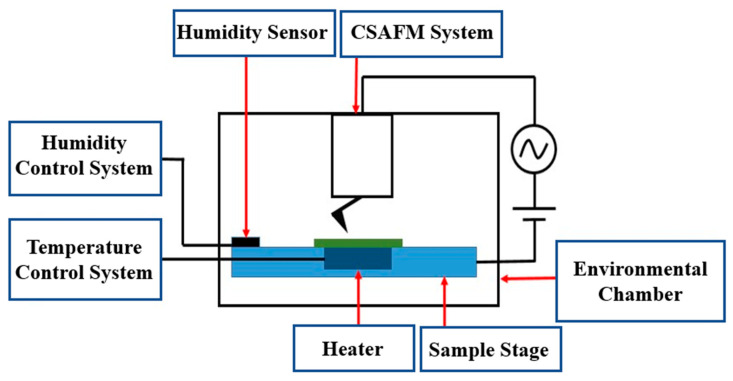
Schematic diagram of the experimental setup for heating and humidity control during CSAFM measurements.

In this study, Nafion 212 was characterized by using CSAFM and its physicochemical characteristics are listed in Table 1. The study was conducted in three steps. The first step was sample preparation and annealing. Annealing was performed at 90 °C for 6–7 h. Nafion 212 membranes were prepared by cutting them into 1 cm × 1 cm pieces. Nafion 212 sample was mounted on the heater, which was attached to the sample stage, in an environmental chamber as shown in Figure 6. In this step, the membrane was heated at 90 °C for 6–7 h. After heating was finished, Nafion 212 was gradually cooled on the heating stage for 2–3 h. After annealing, the environmental chamber was adjusted to maintain a controlled temperature and RH for the subsequent measurements. Second, the morphology and local current of Nafion 212 were simultaneously characterized using CSAFM. The data from morphology and local current of Nafion 212 is analyze by using Gwyddion (v2.41), which is an spm visualization and analysis tool. In the measurement process, a bias voltage of 1.5 V was applied between the Pt-coated tip and a sample holder to map local current. After the measurement was completed, the same membrane was re-annealed while maintaining the same conditions. All measurements were performed at room temperature and 60% RH. Third, the morphology and current maps, which contain height and current information, were analyzed using statistical and numerical methods. In the statistical analysis, frequency distributions were obtained from pixel data in the topography and current maps, which contain height and current information. Histogram peak values and FWHMs were calculated to evaluate trends in ionic channel network variation. A numerical approximation method was also applied to quantify ionic channel network characteristics. Finally, the mechanism of ionic channel network variation was interpreted based on changes in the number of protons moving through the network (NPMI) during continuous annealing at 90 °C.

## 3. Results

**Figure 7:**
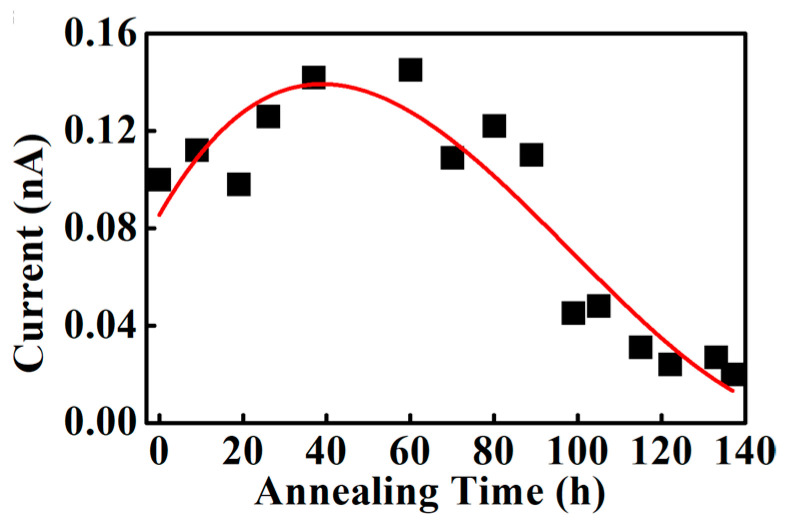
The mean current variation under continuous annealing at 90 °C. Black squares and red curved represent current and reference line, respectively.

Figure 7 shows the variation in mean current for Nafion 212 annealed at 90 °C. The mean current is associated with proton conductivity, as it reflects proton transport through the ionic channel network. When hydrogen, generated by electrolysis, reaches the Pt-coated tip surface, it is oxidized into protons and electrons via the catalytic activity of Pt. The measured current corresponds to the flow of electrons into the sensing circuit and therefore reflects the local surface conductance. From 0 to 60 h of annealing, the mean current increases continuously. The mean current increases from 0.1 nA in the unannealed condition to 0.15 nA after 60 h, corresponding to a 50% increase in surface conductance. This increase is attributed to the morphological reconstruction of Nafion 212 during annealing. Thermal treatment promotes the ionomer reorganization, leading to reduced domain size and enhanced long-range mobility of polymer backbones or ionic aggregates due to thermally induced dispersion [33]. This morphological evolution increases the number of protons moving through the ionic channel network. With continued annealing beyond 60 h, the mean current gradually decreases up to 80 h. After 137 h of annealing, the mean current decreases to 0.02 nA, which is approximately a seven-fold decrease than the maximum value. This trend indicates continuous evolution of the Nafion 212 morphology during prolonged annealing. Between 60 and 137 h, these structural changes reduce proton transport through the ionic channel network. Thus, the morphology of Nafion 212, which governs the ionic channel network, continuously evolves when subjected to prolonged heating slightly above typical operating temperatures.

**Figure 8:**
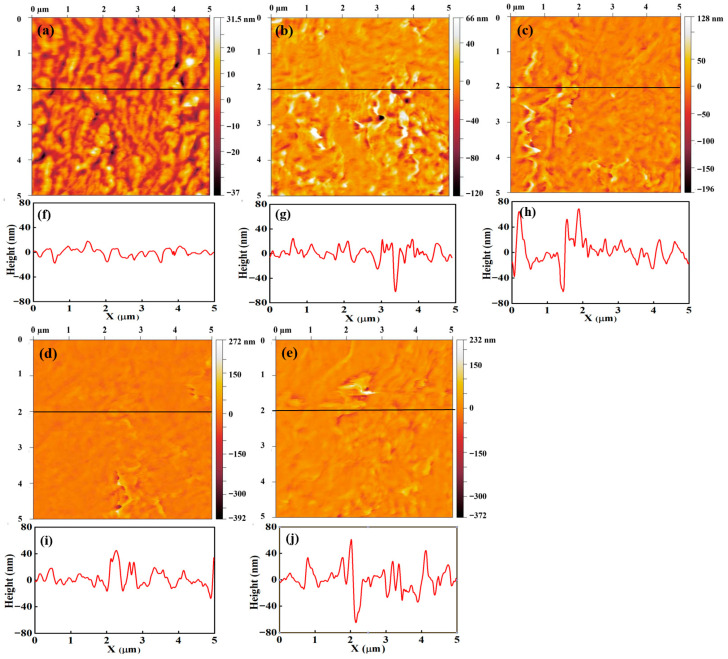
Topography (**a**–**e**) and corresponding line profiles (**f**–**j**) of Nafion 212 under various annealing conditions: (**a**,**f**) unannealed, (**b**,**g**) 26 h, (**c**,**h**) 60 h, (**d**,**i**) 103 h, and (**e**,**j**) 137 h. The black line in the topography indicates the location of the line profile.

To examine morphological changes, topography and current-sensing images of unannealed Nafion 212 and samples annealed for 26, 60, 103, and 137 h were selected for qualitative and quantitative analysis. Figure 8 presents the topography and corresponding line profiles. The color scale represents surface height variation, with brighter regions indicating higher elevations. No clear geometric trend is observed in the surface morphology with increasing annealing time. All images exhibit winding, grooved features, which are more pronounced in Figure 8a–c. In contrast, these features become less distinct in Figure 8d,e. The line profiles represent height variations along selected cross-sections and provide limited insight into overall surface geometry. However, the line profiles reveal an increase in height variation with annealing time. For unannealed Nafion 212, the surface height ranges from approximately −15 to 15 nm, whereas for samples annealed for 26, 60, and 137 h, the height variation increases to approximately −40 to 40 nm. These results indicate that the Nafion 212 surface becomes rougher with annealing.

**Figure 9:**
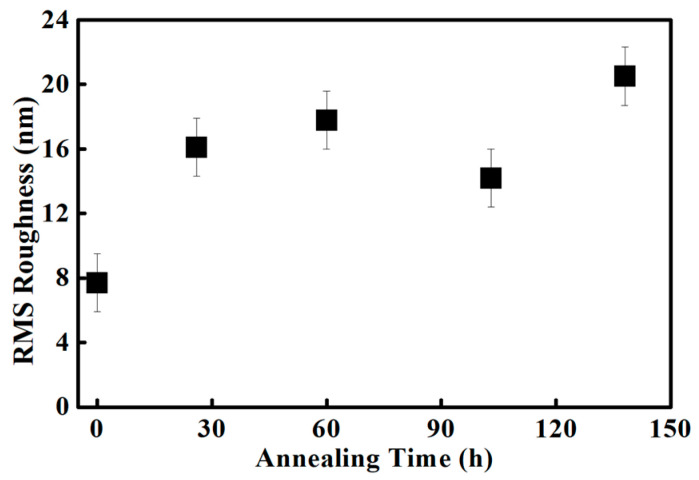
RMS roughness variation in Nafion 212 as a function of annealing time.

The RMS roughness was calculated from each topography image to quantitatively assess geometric variation induced by annealing as shown in Figure 9. Surface roughness represents deviations in surface height relative to the mean plane. The RMS roughness [34] is defined as follows [34]:

(1) $$R_{q}=\sqrt{\frac{1}{n}\sum_{i=1}^{n}{\left(z_{i}-\bar{z}\right)}^{2}},$$

where R_q_, z_i_, and $\bar{z}$ denote the RMS roughness, the height at each pixel, and the average height, respectively. Figure 9 shows the RMS roughness for unannealed Nafion 212 and samples annealed for 26, 60, 103, and 137 h, with values of 7.7, 16.1, 17.8, 14.2, and 20.5 nm, respectively. All annealed samples exhibit approximately twofold higher RMS roughness than unannealed Nafion 212, indicating that annealing induces significant structural changes. These changes are likely to influence Nafion 212 performance. Overall, RMS roughness increases with annealing time, although a slight decrease is observed at 103 h. The rate of increase in RMS roughness decreases with prolonged annealing. Annealing induces surface expansion and wrinkling of Nafion 212, leading to an overall increase in RMS roughness [35]. This expanded surface morphology is retained during subsequent heating and cooling cycles, indicating persistent structural modification [36]. During the initial stage of annealing, the expansion rate is relatively high but decreases with continued annealing. This rapid early-stage expansion significantly alters the geometry of the ionic channel network and polymer backbone. Overall, RMS roughness analysis reveals trends in geometric structural variation induced by annealing. However, it provides only indirect evidence of structural changes in Nafion 212; more detailed characterization of the ionic structure is required to fully understand variations in the ionic channel network.

**Figure 10:**
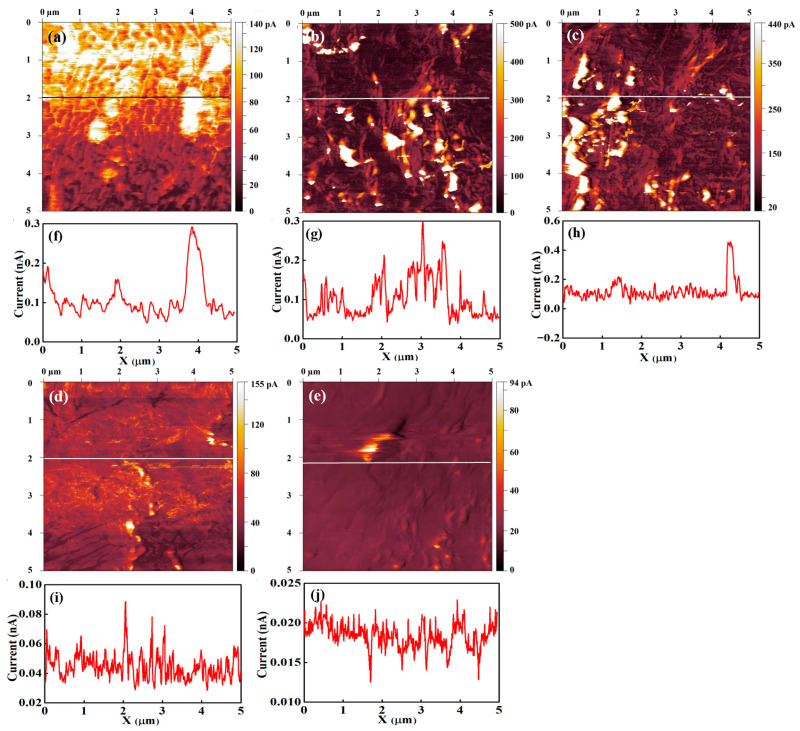
Current-sensing images (**a**–**e**) and corresponding line profiles (**f**–**j**) of Nafion 212 under various annealing conditions: (**a**,**f**) unannealed, (**b**,**g**) 26 h, (**c**,**h**) 60 h, (**d**,**i**) 103 h, and (**e**,**j**) 137 h. The black and white lines in the topography indicate the location of the line profile.

Figure 10 presents current-sensing images and the corresponding line profiles for the unannealed sample and samples annealed for 26, 60, 103, and 137 h. Figure 10a shows the current-sensing image of the unannealed sample. A clear contrast between bright and dark regions is observed, corresponding to relatively high and low current, respectively. The bright regions cover most of the surface and exhibit a broad, winding morphology, while the dark regions are located between them and occupy narrower areas. The current-sensing image of the 60 h annealed sample shows a distinctly different surface morphology. The current values in both the bright and dark regions increase compared with the unannealed condition. The broad, winding high-current features are substantially reduced, and the width of the dark regions also decreases. These results suggest that the current in the dark regions of unannealed Nafion 212 increases upon annealing, likely due to structural changes in the backbone chains or micellar domains. Such structural modifications may also enhance ionic dispersion.

Bright and dark regions are also observed in the current-sensing images of the sample annealed for 103 h. However, the current values in both regions are lower than those of the unannealed and the 26 h and 60 h annealed samples. The boundary between the bright and dark regions becomes indistinct due to the reduced current contrast. In contrast, the current-sensing image of the 137 h annealed sample exhibits a markedly different surface morphology. Distinct bright and dark regions are no longer observed, and the overall current is significantly lower than in all other conditions. At this stage, the current-sensing image appears to reflect the topography, indicating that the ionic structure at the surface is no longer resolved. This behavior suggests that, under conditions of extremely small or diminished ionic clusters, the contact area between the conductive tip and the surface dominates the measured current. The contact area depends on the surface geometry; for example, it is larger on flat regions than on grooved or stepped areas. Consequently, when ionic clusters—responsible for surface conductance—are sparse or reduced in size, the number of clusters in contact with the conductive tip becomes governed primarily by the tip–surface contact area.

The line profiles of the current-sensing images represent the current variation along selected lines on the surface and provide limited quantitative information. Figure 10f–j show the line profiles for the unannealed sample and samples annealed for 26, 60, 103, and 137 h, respectively. Figure 10f corresponds to the unannealed sample. Alternating high- and low-current regions are clearly observed, consistent with the bright and dark areas in the current-sensing image. The high-current values range from approximately 0.10 to 0.19 nA, whereas the low-current values are below ~0.07 nA. The resulting current contrast is approximately 0.03–0.11 nA. Because the measured current is related to proton transport through the ionic channel network, the current at each pixel reflects the local ionic cluster density at the tip–sample contact. Therefore, the clear separation between high- and low-current regions in the line profile indicates a pronounced spatial segregation of ionic cluster density.

Figure 10g shows the line profile for the sample annealed for 26 h. A clear separation between low- and high-current regions is again observed. The low- and high-current values are approximately 0.04–0.07 nA and 0.12–0.20 nA, respectively. The low-current range is comparable to that of the unannealed sample, whereas the high-current values are increased. Figure 10h presents the line profile for the 60 h annealed sample. Alternating high- and low-current regions are still observed; however, the spatial periodicity is higher than in unannealed Nafion 212. The average current level is ~0.1 nA, indicating an overall increase compared with the unannealed condition. In contrast, the current contrast is reduced to approximately 0.02–0.04 nA. These trends can be attributed to structural reorganization during annealing. Regions with initially low ionic channel density are likely to undergo enhanced ionomer redistribution, promoting the formation and connectivity of ionic channels. As a result, the local current—associated with ionic transport—increases, leading to a higher overall current level and a reduced contrast between high- and low-current regions.

Figure 10i shows the line profile for the sample annealed for 103 h. The profile is similar to that of the 60 h annealed sample, exhibiting frequent alternation between high- and low-current regions. However, the average current level is ~0.04 nA, which is lower than those of the unannealed and the 26 h and 60 h annealed samples. The current contrast is below ~0.02 nA, indicating a further reduction with annealing. This behavior suggests that the ionic channel network density becomes more uniformly reduced at this stage. Figure 10j presents the line profile for the 137 h annealed sample. Frequent current fluctuations are still observed, but the overall current level decreases further to ~0.017 nA, significantly lower than in all other conditions. The difference between high- and low-current regions is extremely small. These results indicate a further reduction in the density and/or connectivity of the ionic channel network compared with the 103 h annealed condition.

There are several quantities which affect the surface conductance of Nafion 212 such as contact area between a conductive tip and the sample surface, water content on the surface, and the ionic channel network density. The amount of charge carriers passing through a current sensor is proportional to the contact area for the conducting materials. The surface conductance of Nafion 212 correlates with the number of protons, which move through the ionic channel network. Therefore, the surface conductance of Nafion 212 is proportional to the ionic channel density. Due to the water content in the Nafion 212, a water meniscus forms between the tip and the sample surface. The increase in the water meniscus between a tip and the sample surface leads to an increase in the effective contact area. For constant RH and uniform ionic channel network distribution, the current-sensing images from Nafion 212 simultaneously reflect surface conductance and morphology due to variations in the contact area induced by surface geometry. When the ionic channel network density is high, the influence of surface geometry is minimized because the ionic channel network density under a tip is consistent excluding extremely large surface curvature. When the ionic channel network density is low, the distance between ionic clusters is relatively large. In such case, the number of ionic clusters in contact with the tip is strongly influenced by surface curvature. Thus, surface geometry is dominant under low ionic channel network density. The current-sensing images of un-annealed Nafion 212 reveal the surface’s geometrical features. The winding, grooved features are observed in both topography and current-sensing image as shown in Figure 8a and Figure 10a. These geometrical features in current sensing image are reduced by increase in surface conductance due to continuous annealing. The current sensing image from 137 h annealed condition exhibits extremely low surface conductance, indicating that the ionic channel network density reaches its minimum under prolonged annealing. Thus, the geometrical feature in current-sensing image is dominant and current-sensing image shows resemblance to the topography as shown in Figure 8e and Figure 10e.

The topography, current-sensing images, and line profiles of continuously annealed Nafion 212 reveal pronounced morphological evolution. Annealing increases the surface roughness relative to the unannealed condition. The current-sensing images provide more detailed insight into these changes. The unannealed sample exhibits a clear separation between high- and low-current regions. After annealing for 60 h, the overall surface current increases, while the contrast between high and low currents decreases, consistent with an increase in ionic channel network density associated with structural reorganization. With further annealing beyond 60 h, the overall current decreases, suggesting a reduction in the density and/or connectivity of the ionic channel network. These interpretations of the ionic structural evolution during continuous annealing are qualitative and limited in scope. To obtain a more quantitative and comprehensive understanding, histograms are extracted from each image.

**Figure 11:**
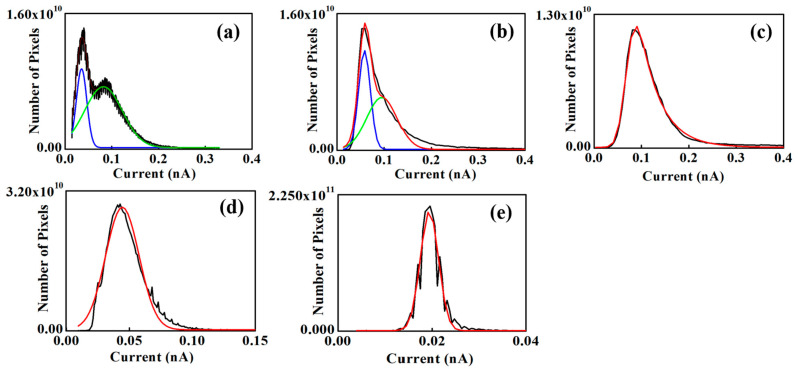
Histograms of (**a**) unannealed, (**b**) 26 h, (**c**) 60 h, (**d**) 103 h, and (**e**) 137 h annealed Nafion 212. The blue and green lines in (**a**) and (**b**) indicate the Gaussian fitting of the first and second peaks of the multi-peaked histogram, respectively. The red line indicates the Gaussian fitting of the single-peaked histogram.

Figure 11a–e show the histograms for the unannealed sample and samples annealed for 26, 60, 103, and 137 h, respectively. The histograms for the unannealed and 26 h annealed samples exhibit bimodal distributions, consisting of a sharp peak at low current and a broader peak at relatively higher current (Figure 11a,b). In contrast, the histograms for the 60–137 h annealed samples display approximately normal (Gaussian-like) distributions with a single, sharper peak. The distribution width decreases with increasing annealing time beyond 60 h, indicating a reduced current dispersion. The peak position represents the most probable current value and is related to the dominant ionic transport pathways, which are associated with the ionic channel network in Nafion 212. The FWHM reflects the spread of current values across the image. A narrower FWHM indicates more uniform current distribution; if the ionic channel network is spatially homogeneous, most pixel currents cluster near the mean value, resulting in a sharper histogram. A non-uniform distribution of pixel currents produces a broader histogram. For the unannealed sample, two peaks are observed at approximately 0.03 and 0.08 nA. The lower-current peak corresponds to regions with relatively low ionic channel density (dark contrast in the current-sensing image), whereas the higher-current peak corresponds to regions with higher ionic channel density (bright contrast). The presence of two distinct peaks provides clear evidence of spatially non-uniform ionic channel network density. For the 26 h annealed sample (Figure 11b), a pronounced bimodal distribution is also observed, with peaks at approximately 0.06 and 0.10 nA—both higher than those of the unannealed condition. The shift in the lower-current peak is larger than that of the higher-current peak, suggesting a more substantial increase in ionic channel density (or improved transport) in the initially low-current regions compared with the high-current regions. All annealed conditions, except the 26 h sample, exhibit approximately normal (Gaussian-like) distributions. The peak current values for the 60 h, 103 h, and 137 h annealed samples are ~0.07, 0.04, and 0.02 nA, respectively. With annealing (beyond 26 h), the histograms evolve from bimodal to unimodal distributions, indicating increased uniformity of the ionic channel network, likely due to enhanced ionic dispersion. The decrease in peak current from 60 to 137 h suggests a progressive reduction in the density and/or connectivity of the ionic channel network. This reduction in proton conductivity may be associated with rearrangement of water channels, such as narrowing or disconnection, arising from changes in hydrophilic and hydrophobic domain organization during annealing [30].

The FWHM values for the unannealed sample are ~0.02 and ~0.08 nA for the first and second peaks, respectively. The second peak is therefore approximately fourfold broader than the first. This difference is attributed to the larger spatial extent of the bright (high-current) regions associated with the second peak, compared with the narrower dark (low-current) regions corresponding to the first peak. For the 26 h annealed sample, the FWHM values of the first and second peaks are ~0.03 and ~0.08 nA, respectively. Both peaks shift slightly toward higher current values, while their widths remain largely unchanged. For the 60 h, 103 h, and 137 h annealed samples, the FWHM values are ~0.07, 0.03, and 0.005 nA, respectively. A pronounced decrease in FWHM is observed beyond 60 h of annealing. Since FWHM reflects the dispersion of current values across pixels—and is therefore related to the spatial variation in ionic channel network properties—this reduction indicates increasing uniformity of the ionic channel network with prolonged annealing. The increase in uniformity of the ionic channel network from 0 to 60 h is attributed to enhanced ionic dispersion arising from structural reorganization of the backbone chains or ionic domains (often described as micellar/clustered structures). This interpretation is supported by the increase in mean current in Nafion 212. From 60 to 137 h, the continued increase in uniformity is accompanied by a reduction in the overall ionic channel network density, likely due to degradation or disruption of the network. Prolonged annealing of Nafion 212 has been reported to cause performance deterioration, associated with disconnection of water channels between sulfonic acid groups or increased surface hydrophobicity [30]. The continuous decrease in FWHM suggests that the reduction in ionic channel density occurs more prominently in initially high-density (high-current) regions. As a result, the spatial distribution of ionic channel density becomes more uniform with extended annealing.

## 4. Discussion

In a previous study, we proposed NPMI for the quantitative analysis of the ionic channel network based on electrodynamic principles and CSAFM [37]. NPMI was formulated under the following assumptions: (i) proton transport within the membrane is analogous to electron flow through an external circuit, and (ii) protons reach a steady state during tip scanning within the ionic channels. Under these conditions, NPMI provides information on the total ionic channel network density beneath the Pt-coated tip. If the number of ionic clusters in contact with the tip can be estimated, a more detailed insight into the ionic channel network can be obtained. Specifically, the ratio of NPMI to the number of contacting ionic clusters yields the number of protons transported through an ionic channel network associated with a single cluster. This quantity reflects proton transport along an individual conduction pathway in Nafion 212. Therefore, this approach enables the evaluation of both ionic channel density and single-path variation under annealing.

NPMI was derived from the fundamental principles of electrodynamics and CSAFM. NPMI was defined as [37]:

(2) $$N=\frac{I}{qn_{p}v_{s}}.$$

where *I*, *q*, n_p_, and *v_s_* are the current value, the elementary charge, the number of pixels per line, and the scan rate, respectively.

The NPMI values for unannealed and annealed Nafion 212 were calculated using numerical approximation. Each CSAFM image was acquired at a 1 Hz scan rate, meaning that the scan time for one line is 1 s, with 512 pixels per line. The elementary charge, q, was taken as 1.6 × 10^−19^ C. The resulting NPMI values are summarized in Table 2.

**Figure 12:**
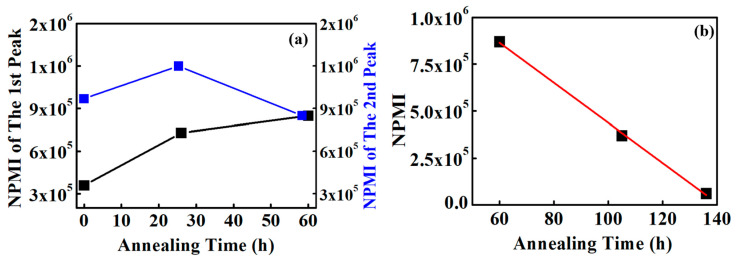
Variations in NPMI from (**a**) unannealed to 60 h and (**b**) 60 h to 137 h, based on peak values from the histograms. The blue and black lines in (**a**) indicate the NPMI variations of the first and second peaks based on the histogram from the unannealed to the 60 h annealed condition, respectively. The red line in (**b**) indicates the NPMI variation from the 60 h to the 137 h annealed condition.

The NPMIs for all unannealed and annealed conditions were calculated from the peak current values to compare representative ionic channel networks in each Nafion 212 sample as shown in Figure 12. Unannealed Nafion 212 exhibits two peaks, yielding two NPMI values: 2.5 × 10^5^ for the low-current region and 9.7 × 10^5^ for the high-current region. This result reflects a clear distinction between regions of low and high ionic channel density. NPMI represents the number of protons simultaneously moving through the ionic channel network and, therefore, directly reflects ionic channel density. The difference between the two NPMI values in the unannealed sample indicates local variations in ionic channel density. For the 26 h annealed sample, the two NPMIs are 7.3 × 10^5^ and 1.2 × 10^6^. The first peak increases by a factor of 2.7 compared with the unannealed condition, indicating a substantial increase in ionic channel density in regions that initially exhibited low current. For the 26 h annealed sample, the second peak shows only a slight increase compared with the high-current peak of the unannealed sample. Analysis of the changes in the first and second peaks during early annealing indicates that the ionic channel density in the initially low-current regions is significantly enhanced. For the 60 h annealed sample, the NPMI is 8.5 × 10^5^. This value is larger than the first peak of the unannealed sample and slightly smaller than the second peak of both the unannealed and 26 h annealed samples. The ratio of the first peak between the unannealed and 60 h annealed samples is 2.4, indicating that ionic channel density in the low-current regions increases due to enhanced ionic dispersion. The ratio for the second peak is 0.9, suggesting a slight reduction in the high-current regions, also influenced by ionic dispersion. These results indicate that the ionic channel network density increases in initially low-density regions and decreases slightly in high-density regions (Figure 10a). Consequently, the ionic channel network becomes more uniform through a combination of substantial enhancement in low-density regions and minor reduction in high-density regions driven by increased ionic dispersion.

The NPMIs for the 103 h and 137 h annealed Nafion 212 samples are 3.7 × 10^5^ and 6.1 × 10^4^, respectively, indicating a continuous decrease in NPMI after 60 h. The ratio of NPMI between 103 h and 60 h annealed Nafion 212 is 0.43, suggesting that the available proton conduction pathways are reduced by more than 50%, which correlates directly with the observed performance drop of Nafion 212. Since decomposition of the sulfonic acid groups occurs only above 200 °C, and the nano-PEMFC operates for a very short duration, neither thermal degradation nor hydroperoxide attack is likely responsible for the performance decline. Therefore, the reduction in NPMI is attributed to structural variations in the ionic channel network, reflecting a decrease in ionic channel density. Considering the ratio between the 103 h and 60 h annealed samples, the ionic channel density at 103 h is approximately 57% lower than at 60 h. The NPMI of the 137 h annealed Nafion 212 sample is 6.1 × 10^4^, and the ratio of NPMI between the 137 h and 103 h samples is 0.16. This indicates that the NPMI of the 137 h sample is reduced by 84% compared with the 103 h annealed sample. Accordingly, the ionic channel network density is also reduced by approximately 84% relative to the 103 h condition. From 60 to 137 h, NPMI decreases continuously and nearly linearly, suggesting a linear reduction in ionic channel network density due to structural variations, as illustrated in Figure 12b.

**Figure 13:**
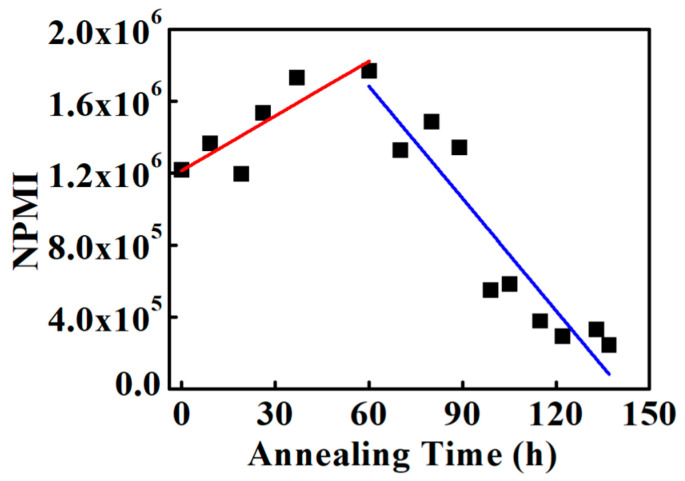
NPMI from average current-sensing value under continuous annealing. The red and blue lines are the fitting lines of NPMI based on the average current-sensing values from the unannealed to 60 h annealed condition, and from the 60 h to 137 h annealed condition, respectively.

Understanding ionic channel network variation based on NPMI calculated from histogram peak values is challenging because early annealing conditions exhibit multiple peaks, unlike the 60–137 h annealed samples. To obtain a more systematic and comprehensive view, the NPMI was calculated using the mean current at each annealing time as shown in Figure 13. From the unannealed condition to 60 h annealing, NPMI increases approximately linearly as shown in Figure 13. The NPMI values for the unannealed and 60 h annealed samples are 1.2 × 10^6^ and 1.7 × 10^6^, respectively, representing a nearly 50% increase. Assuming a linear increase, the NPMI growth rate from the unannealed condition to 60 h annealing is approximately 1 × 10^4^ ± 3 × 10^2^ h^−1^. Table 3 shows performance variation by annealing from this research and other studies. It is difficult to directly compare the performance variation by annealing between this study and other studies because this work utilizes NPMI for characterization, while others use proton conductivity. Nevertheless, it is possible to compare performance trends. Annealing conditions such as temperature and times are not consistent for all studies; however, all studies show an increase in performance following appropriate annealing. Nafion assistance of 3,4-dimethylbenzaldehyde [38], which is done under enhanced/accelerated annealing conditions to enhance the annealing effect, exhibits 75% performance enhancement. It is comparable to the result of this study.

After 60 h of annealing, NPMI begins to gradually decrease. NPMI calculated from the mean current between 60 and 137 h is consistently higher than the NPMI derived from the peak values at the same annealing times. For the 137 h annealed sample, the NPMI is 2 × 10^5^, approximately tenfold smaller than the NPMI of the 60 h annealed sample, which represents the maximum value. This indicates that the ionic channel density at 137 h is reduced by a factor of 10 relative to the 60 h condition. Assuming a linear decrease between 60 and 137 h, the estimated rate of NPMI reduction is approximately 1.9 × 10^5^ ± 8 × 10^2^ h^−1^.

## 5. Conclusions

In this study, the variation in the ionic channel network in Nafion 212 under continuous annealing was investigated using CSAFM combined with a numerical approximation method. The membrane was analyzed qualitatively and quantitatively using RMS roughness from topography images and histograms from current-sensing images. Upon annealing, RMS roughness increased compared with unannealed Nafion 212. Histograms revealed two peaks from the unannealed sample up to 26 h of annealing, which merged into a single peak between 60 and 137 h. The peak current value decreased continuously from 60 to 137 h, while the FWHM, representing the dispersion of pixel currents, gradually narrowed over the same interval. During early annealing, the ionic channel network density increased due to structural reorganization, reducing local differences in ionic channel density across pixels. Beyond 60 h, the ionic channel network density decreased continuously, reflecting progressive decomposition or disruption of the network.

NPMIs were calculated from the histograms to assess the ionic channel network density. For the unannealed and 26 h annealed samples, two NPMI values were observed corresponding to the low- and high-current regions, indicating an uneven distribution of ionic channel density in Nafion 212. With annealing, the two NPMI values merged into a single value: the first peak increased while the second peak decreased, representing the formerly low- and high-current regions, respectively. This indicates that annealing enhances the ionic channel network density in low-current regions while reducing local differences, leading to a more uniform distribution. After 60 h, the ionic channel network begins to decrease linearly. To quantify this, NPMI was also calculated from the mean current of each current-sensing image. From the unannealed condition to 60 h, the ionic channel network density increases linearly, with a rate of approximately 1 × 10^4^ h^−1^ ± 3 × 10^2^ h^−1^. Beyond 60 h, the network density decreases linearly, with a rate of approximately 1.9 × 10^5^ h^−1^ ± 8 × 10^2^ h^−1^.

The study focuses on the ionic channel network density using NPMI. NPMI value provides an indirect measure for characterizing the ionic structure of the proton exchange membranes. It is based on the pixels of the surface current, which are correlated with the tip contact area. Consequently, the NPMI allows for the analysis of a relatively large area compared to individual clusters; this is because the tip contact area is approximately 100 nm, whereas an ionic cluster is only 2–3 nm. In future studies, it will be necessary to further refine this model to enable a more precise analysis of channel variations mediated by a single cluster. In addition, various other types of PEMs will be characterized using the refined model.

## Figures and Tables

**Table 1:** Physicochemical characteristics of Nafion 212.

Property	Unit	Typical Value (Typical)
Thickness	μm (mil)	50.8 (2.0)
Equivalent Weight	g/eq	1100
Density	g/cm^3^	1.97
Ion Exchange Capacity	meq/g	0.91
Water Uptake	%	38 ± 5
Proton Conductivity	Scm^−1^	>0.10 (at 80 °C, 100% RH)
Tensile Strength	MPa	23–32 (at 23 °C, 50% RH)

**Table 2:** NPMI of continuous annealing based on a peak value from the histogram.

Conditions	Unannealed		26 h Annealed		60 h Annealed	103 h Annealed	137 h Annealed
	1st Peak	2nd Peak	1st Peak	2nd Peak			
NPMI	2.5 × 10^5^	9.7 × 10^5^	7.3 × 10^5^	1.2 × 10^6^	8.5 × 10^5^	3.7 × 10^5^	6.1 × 10^4^

**Table 3:** Comparison of performance variation under various annealing conditions.

	Annealing Condition	Before Annealing	After Annealing	References
Nafion 212	90 °C, 60 h	1.22 × 10^6^ (NPMI)	1.77 × 10^6^ (NPMI)	This study
Nafion (assistance of 3,4-dimethylbenzaldehyde))	80 °C, 8 h	7.9 × 10^−2^ Scm^−1^	1.3 × 10^−1^ Scm^−1^	[38]
Nafion	140 °C, 6 h	2.5 × 10^−3^ Scm^−1^	8.2 × 10^−3^ Scm^−1^	[39]
Nafion^®^ membrane	165 °C, 3 h	1.2 × 10^−2^ Scm^−1^	9.0 × 10^−2^ Scm^−1^	[29]
Nafion 117	210 °C, 1 h	1.0 × 10^−1^ Scm^−1^	1.3 × 10^−1^ Scm^−1^	[40]

## Data Availability

The original contributions presented in this study are included in the article. Further inquiries can be directed to the corresponding author.

## Abbreviations

The following abbreviations are used in this manuscript:

AEM	Anion-exchange membrane
AFM	Atomic force microscopy
CSAFM	Current-sensing atomic force microscopy
EFM	Electrostatic force microscopy
FWHM	Full width at half maximum
IEC	Ion exchange capacity
PEM	Proton-exchange membrane
PEMFC	Proton-exchange membrane fuel cell
RH	Relative humidity
RMS	Root mean square
NPMI	Number of protons moving through an ionic channel network
Variables	
*R_q_*	Root mean square (RMS) roughness
*z_i_*	Local height at the i-th pixel
$\bar{z}$	Arithmetic average height
*I*	Current measured via CSAFM
*q*	Elementary charge
*n_p_*	Number of pixels per line
*v_s_*	Scan rate of CSAFM
